# Synchronous Gastrointestinal Tumor and Abdominal Aortic Aneurysm or Dissection Treated with Endovascular Aneurysm Repair Followed by Tumor Resection

**DOI:** 10.1155/2019/8087256

**Published:** 2019-01-06

**Authors:** Bo Zhang, Ketong Wu, Yang Liu, Haiyang Lai, Zhaofei Zeng

**Affiliations:** Department of Radiology, The Sixth Affiliated Hospital of Sun Yat-sen University, Guangzhou 510655, China

## Abstract

**Objective:**

To evaluate the strategy in the management of patients with synchronous gastrointestinal tumor and abdominal aortic aneurysm (AAA) or abdominal aortic dissection (AAD) undergoing endovascular repair followed by tumor resection.

**Materials and Methods:**

Five patients with synchronous gastrointestinal tumor and AAA or AAD were treated by endovascular repair followed by tumor resection. Clinical data were retrospectively analyzed with respect to the management strategy, safety, and outcome.

**Results:**

Endovascular repair was technically successful in all patients. All the stents were well positioned and well patent, and the AAA (*n* = 3) or AAD (*n* = 2) were correctly excluded without endoleaks. After endovascular repair, all patients underwent resection of gastrointestinal tumor. No late mortality or major complications related to the two procedures were observed in the subsequent follow-up.

**Conclusion:**

Our results demonstrate that EVAR could significantly shorten the delay between AAA and gastrointestinal procedure with an excellent postoperative outcome. If the anatomical criteria are satisfied, EVAR followed by tumor resection might be an effective treatment for concomitant AAA and gastrointestinal tumor.

## 1. Introduction

As the aging population increases, the incidence of gastrointestinal cancer and vascular diseases, such as abdominal aortic aneurysm (AAA) and abdominal aortic dissection (AAD), has increased [1, 2]. Though the true incidence is difficult to accurately ascertain, the synchronous presentation of AAA or AAD and gastrointestinal tumor is still rare (0.49% to 2.1% in most series [3, 4]). In the absence of a clear consensus from the literatures, the management of this clinical presentation remains perplexing and controversial, especially in establishing the optimal treatment approach and the benefit of the patients [1, 3].

To resect gastrointestinal tumor first may increase the risk of rupture of AAA or AAD in the perioperative period. Conversely, AAA or AAD repair before gastrointestinal tumor resection may result in the delay of some cancer therapy and consequent cancer progression [5, 6]. But the availability of endovascular techniques for AAA, AAD, and aortoiliac aneurysm repair has significantly shortened the delay between aneurysm repair and gastrointestinal tumor surgery, which may add new options for the complex therapeutic paradigm [7, 8].

The aim of this study was to evaluate the strategy in the management of patients with synchronous gastrointestinal tumor and AAA or AAD undergoing endovascular repair followed by tumor resection.

## 2. Materials and Methods

### 2.1. Clinical Material

Between October 2014 and September 2017, 5 cases with synchronous gastrointestinal tumor and AAA or AAD were treated by endovascular repair followed by cancer resection. AAA or AAD was detected at a routine preoperative CT examination. The anatomical criteria should include a healthy part of the infrarenal aorta (neck) with a diameter not exceeding 30 mm and a length of at least 15 mm, a neck angulation of <60°, absence of thrombus, or calcifications disposed circumferential in the aneurysm neck or the iliac arteries [6]. The study protocol was approved by the Institutional Ethics Review Board of our hospital. Written informed consent prior to treatment was obtained from each patient.

### 2.2. Procedures

Endovascular repair was performed under local infiltration anesthesia in all 5 cases. Both transfemoral puncture approaches were used for artery access. To minimize the risk of colorectal ischemia, at least unilateral hypogastric artery was preserved in all the endovascular procedures. All the repairs were performed using Endurant Stent Graft System (Medtronic Inc., Santa Rosa, CA, USA). In addition, 3 stent grafts were used with an iliac branch device to treat 3 associated iliac artery aneurysms. Before and after endovascular repair, angiographies were performed to confirm the deployment of stent grafts, the complete exclusion of AAA or AAD, the patency of the renal and hypogastric arteries, and no endoleak. And then, the femoral punctures of all patients were treated with vascular closure devices (Perclose ProGlide, Abbott, USA) to suture the puncture of both common femoral arteries.

One patient (case 1) with rectal cancer underwent neoadjuvant chemotherapy (FOLFOX regimen, 3 courses) after AAA endovascular repair and was operated of rectal cancer after 115 days. The other 4 patients underwent a resection of gastrointestinal tumor by means of laparoscopic procedure within 2 weeks after endovascular repair of AAA (*n* = 2) or AAD (*n* = 2). All tumors were staged according to the TNM classification system.

### 2.3. Data Analysis and Follow-Up

Clinical records were analyzed retrospectively with respect to tumor type and stage, endovascular repair, surgical treatment of gastrointestinal tumor, sequential therapy, perioperative complications, and overall outcome, and they were collected from the original hospital charts, operation notes, and outpatient medical records via telephone questionnaires.

## 3. Results

### 3.1. Clinical Characteristics

A total of 5 patients (all male) with gastrointestinal tumor and AAA or AAD undergoing endovascular repair followed by tumor resection were included in our study. The patient age ranged from 61 to 79 years with a mean age of 68.4 ± 6.7 years. Clinical characteristics of these patients were summarized in Table 1.

### 3.2. Technique and Clinical Outcomes

**Figure 1:**
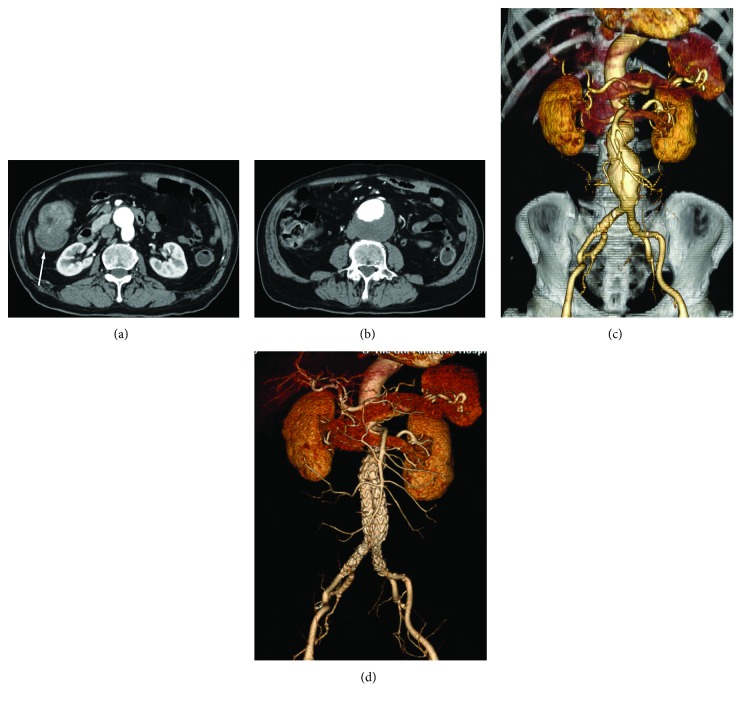
A 79-year-old man with synchronous abdominal aortic aneurysm (AAA) and right colon cancer (a, arrow). Contrast-enhanced CT scan revealed an infrarenal AAA that measured 68 mm in maximum diameter with mural thrombus (b). Preoperative endovascular repair 3D angiogram revealed an infrarenal abdominal aortic aneurysm (c). Postoperative 3D angiogram confirmed a patent endovascular graft in a good position with the preservation of bilateral hypogastric arteries (d).

**Figure 2:**
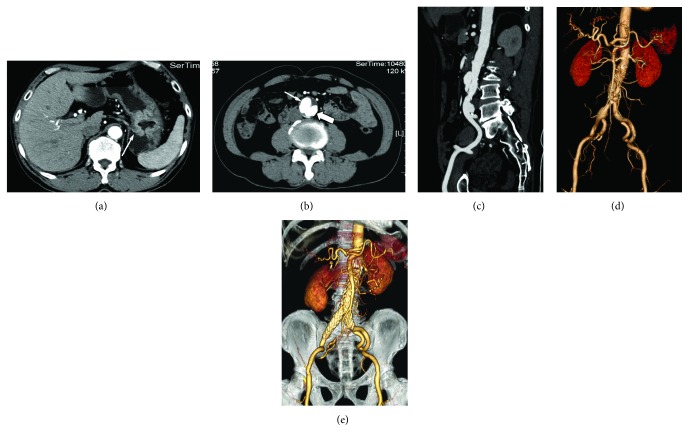
A 61-year-old man with synchronous abdominal aortic dissection (AAD) and gastric cancer (a, arrow). Contrast-enhanced CT scan showed AAD with true lumen (thick arrow) and false lumen (thin arrow) (b). The thin layer MIP image (c) and 3D angiogram (d) revealed an infrarenal AAD and right common iliac aneurysm (25 mm in diameter). Postoperative 3D angiogram confirmed a patent endovascular graft in a good position without endoleaks (e).

Endovascular repair and suture of the puncture of femoral arteries were technically successful in all patients (Figure 1). All the stents were well positioned and well patent, and the aneurysms or dissections were correctly excluded on contrast-enhanced CT scans performed between 2 and 7 days after the endovascular procedure (Figure 2). No complication related to the endovascular stent or at the site of femoral artery puncture was observed.

After endovascular repair, all patients underwent resection of gastrointestinal tumor. The patient (case 1) with rectal cancer was treated from open surgery after neoadjuvant chemotherapy (FOLFOX regimen, 3 courses). The other 4 patients were treated by means of laparoscopic procedure within 2 weeks after endovascular repair.

During a mean follow-up of 21 months (median 20 months; range 3–44 months), two patients underwent adjuvant chemotherapy, and no special treatment was needed for the other 3 patients (Table 2). No late mortality or major complications related to the two procedures were observed in the subsequent follow-up.

## 4. Discussion

The coexistence of AAA or AAD and gastrointestinal tumor is uncommon but may be increasing in incidence with an aging population. The management of this clinical situation remains a therapeutic dilemma, as evidenced by the disparity in published case reports, series, and recommendations [1, 9–11]. Many variables in the decision-making process should be considered, including surgeon's experience and preference, local medical level, aneurysm or dissection size, and type and stage of cancer [12, 13].

In recent years, there has been an increased awareness on surgical treatments with minimal invasiveness for various diseases. In the treatment of AAA or AAD, endovascular repair is a promising alternative to the conventional open graft replacement [14–16]. In a large randomized trial, endovascular abdominal aortic aneurysm repair was associated with a significantly lower 30-day operative mortality (1.8% vs. 4.3%), shorter hospital stay and operation time, and minimal blood loss rate than open repair of AAA [17].

In the absence of such level of evidence, the management of gastrointestinal tumor has evolved into a multidisciplinary effort that requires chemotherapy, radiation, and surgical oncologists to elucidate a sound clinical treatment plan [18]. In order to reduce the probability of recurrence and to improve overall survival, preoperative chemoradiation and then radical excision is currently the standard of care for locally advanced stages of rectal cancer [19, 20]. Similarly, preoperative chemoradiation increases the likelihood of achieving favourable histopathological features that correlate with a 5-year OS > 70% in gastric cancer patients, which might eventually impact long-term outcome [21, 22]. Whereas, for the fear of precipitating aneurysmal rupture, medical oncologists were very reluctant to perform neoadjuvant chemoradiation with an unrepaired AAA [23, 24]. Therefore, endovascular repair of AAA or AAD first may allow safe performance of neoadjuvant chemoradiation therapy for downstaging of gastrointestinal cancer [2, 23]. After 115 days of endovascular repair, one patient (case 1) with rectal cancer was treated from open surgery with neoadjuvant chemotherapy (FOLFOX regimen, 3 courses). The mean delay between endovascular repair and resection of gastrointestinal tumor for the other 4 patients was of 12.5 days (8-14 days), which would have no impact on the oncologic status of the patient.

However, for some anatomical reasons, including too short aneurysmal neck, tortuous arteries, and associated iliofemoral stenoses or obstructions, endovascular repair may not be always feasible and open aneurismal repair should be considered [23]. In addition, one important issue concerning endovascular repair of AAA or AAD in the setting of colorectal cancer is the covering of the inferior mesenteric artery (IMA), which may induce a potential reduced perfusion of the rectum and the sigmoid, especially at the time of a right colectomy for the interruption of the Drummond's arcade [23, 25, 26]. For this reason, it is very important that at least unilateral hypogastric artery should be preserved to minimize the risk of colorectal ischemia. In our study, one patient (case 2) had a right colon adenocarcinoma and underwent endovascular repair with the preservation of bilateral hypogastric arteries followed by laparoscopic resection of right colon cancer, and there was no anastomotic leakage and other major complications related to the two procedures during the 30 months of follow-up. Although endovascular repair should be considered as a valid alternative technique to open vascular surgery for the concomitant aneurysmatic disease and tumor, this treatment is not always feasible in all cases with a normal life expectancy [12].

Based on the small number of our series, endovascular repair followed by tumor resection might be recommended for the synchronous presentation of AAA or AAD and gastrointestinal tumor. In order to explore the strategy more comprehensively, a brief review of the published literature was performed. By searching the terms (“colorectal cancer” or “gastric cancer” or “gastrointestinal tumor”) and (“abdominal aortic aneurysm” or “abdominal aortic dissection”), the related literatures in English language within the recent ten years via the PubMed database were analyzed. As shown in Table 3, a total of 59 patients were identified and analyzed based on the management strategy and outcome, which demonstrated that 42 patients (71.2%) were treated by EVAR and the other 17 patients (28.8%) by open surgery for AAA. In EVAR group, the patients with EVAR first, cancer resection first, or synchronous treatment were 25 (59.5%), 7 (16.7%), and 10 (23.8%), respectively. On the whole, EVAR followed by cancer resection might be an effective management for synchronous abdominal aortic aneurysm and gastrointestinal cancer.

There are several limitations in this study. First, the data were analyzed retrospectively, and as such, the study is subject to the inherent limitations of retrospective studies. Second, due to the rarity of the synchronous presentation of gastrointestinal tumor and AAA or AAD, this single-center, observational, preliminary study suffered from a small sample size and short-term follow-up.

In summary, based on the present results and a brief review of the literature, EVAR could significantly shorten the delay between the AAA and gastrointestinal cancer procedure with an excellent postoperative outcome. If the anatomical criteria are satisfied, EVAR followed by cancer surgery might be an effective management for concomitant AAA and gastrointestinal cancer.

## Figures and Tables

**Table 1:** Clinical characteristics of 5 patients with synchronous gastrointestinal tumor and AAA or AAD.

Case no.	Gender/age (years)	Pathological type, stage	AAA diameter, AAD
1	M/68	Rectal adenocarcinoma; T3N1bM0	AAA (52 mm)
2	M/79	Colon adenocarcinoma; T3N0M0	AAA (68 mm)
3	M/65	Rectal adenocarcinoma; T3N2aM0	AAD
4	M/61	Gastric adenocarcinoma; T3N2	AAD and right common iliac aneurysm (25 mm)
5	M/69	Rectal villous adenoma	AAA (50 mm) and bilateral common iliac aneurysm (right 40 mm; left 34 mm)

AAA: abdominal aortic aneurysm; AAD: abdominal aortic dissection.

**Table 2:** Follow-up of 5 patients after endovascular repair and surgical treatment.

Case no.	Endovascular repair	Surgical treatment	Interval (days)^∗^	Treatment during the interval	Sequential therapy	Follow-up (months)	Outcome
1	Aortobiiliac stent graft	Resection of rectal cancer from open surgery	115	Neoadjuvant FOLFOX ^∗^ 3 courses	DEGRAMONT ^∗^ 3 courses; MWA for liver metastasis	44	Survival
2	Aortobiiliac stent graft	Laparoscopic resection of right colon cancer	14	NA	NA	30	Survival
3	Aortobiiliac stent graft	Laparoscopic resection of rectal cancer	8	NA	FOLFIR ^∗^ 3 courses	20	Survival
4	Aortobiiliac stent graft	Laparoscopic total gastrectomy	14	NA	NA	9	Survival
5	Aortobiiliac stent graft	Laparoscopic resection of rectal cancer	14	NA	NA	3	Survival

NA: not available; MWA: microwave ablation.

**Table 3:** Literatures related to endovascular repair followed by tumor resection for the synchronous presentation of AAA and gastrointestinal tumor.

Study, year	Recruitment period	Number of patients	Management strategy	Key outcome
Shalhoub et al., 2009 [1]	2001-2006	13	Cancer resection prior to open aneurysm repair (*n* = 3) or EVAR (*n* = 7); EVAR prior to cancer resection (*n* = 2); synchronous surgery (*n* = 1)	No interval AAA ruptures, graft infection, or postoperative mortalities
Ward et al., 2009 [5]	2009	2	Retroperitoneal aortic repair prior to cancer resection (*n* = 2)	Endovascular stenting was not feasible. Aortic repair followed by an ultralow anterior resection
Minicozzi et al., 2010 [10]	2005-2008	1	Synchronous EVAR and right colectomy	After neoadjuvant TIPS for portal hypertension, 1-stage EVAR and laparoscopic right colectomy
Yoshinaga et al., 2011 [9]	2011	1	Simultaneous EVAR and total gastrectomy	Despite higher operative risk, the patient had a good outcome
Spanos et al., 2011 [2]	2004-2010	5	EVAR first (*n* = 2); synchronous EVAR and cancer resection (*n* = 3)	In 2 patients, EVAR followed by staged CRC resection. In 3 patients, single-stage procedures were performed
Eliescu and Brătucu, 2012 [6]	2012	2	EVAR first (*n* = 1); EVAR for rupture of AAA four years ago (*n* = 1)	An expectation tactic was opted for the high risk of the aneurismal sac operation in the second patient
Matsuno et al., 2012 [12]	2012	1	Synchronous EVAR and distal gastrectomy	EVAR is a safe and effective treatment for high-risk patients
Illuminati et al., 2013 [23]	2001-2011	16	EVAR followed by colectomy (*n* = 16)	EVAR significantly shortened the delay between the two treatments, with an excellent postoperative outcome
Amato et al., 2014 [4]	2009-2012	2	EVAR followed by resection during the same operation (*n* = 2)	No major complications related to the two minimally invasive procedures
Kawai et al., 2015 [7]	2015	1	EVAR followed by laparoscopic sigmoidectomy	Staged treatment of EVAR followed by laparoscopic colectomy may be a promising strategy
Matsumoto et al., 2015 [8]	1990-2012	14	One-stage EVAR and gastric resection (*n* = 4); open AAA repair and gastric resection (AAA first, *n* = 1; cancer first, *n* = 4; simultaneous, *n* = 5)	One-stage procedure including EVAR and gastric resection is feasible
López Arquillo et al., 2017 [11]	2017	1	Stent for acute intestinal obstruction; EVAR followed by cancer resection	No complications; 3-month follow-up

AAA: abdominal aortic aneurysm; AAD: abdominal aortic dissection; EVAR: endovascular aortic repair.

## Data Availability

The data used to support the findings of this study are available from the corresponding author upon request.
